# Wee1 Inhibition Enhances the Anti-Tumor Effects of Capecitabine in Preclinical Models of Triple-Negative Breast Cancer

**DOI:** 10.3390/cancers12030719

**Published:** 2020-03-19

**Authors:** Todd M. Pitts, Dennis M. Simmons, Stacey M. Bagby, Sarah J. Hartman, Betelehem W. Yacob, Brian Gittleman, John J. Tentler, Diana Cittelly, D. Ryan Ormond, Wells A. Messersmith, S. Gail Eckhardt, Jennifer R. Diamond

**Affiliations:** 1 Division of Medical Oncology, Department of Medicine, University of Colorado Anschutz Medical Campus, 12801 E 17th Ave, Aurora, CO 80045, USA; Dennis.Simmons@childrenscolorado.org (D.M.S.); stacey.bagby@cuanschutz.edu (S.M.B.); Sarah.j.hartman@cuanschutz.edu (S.J.H.); betelehem.yacob@cuanschutz.edu (B.W.Y.); Brian.gittleman@cuanschutz.edu (B.G.); John.tentler@cuanschutz.edu (J.J.T.); wells.messersmith@cuanschutz.edu (W.A.M.); Jennifer.diamond@cuanschutz.edu (J.R.D.); 2 Department of Pathology, University of Colorado Anschutz Medical Campus, 12801 E 17th Ave, Aurora, CO 80045, USA; DIANA.CITTELLY@CUANSCHUTZ.EDU; 3 Department of Neurosurgery, University of Colorado Anschutz Medical Campus, 12801 E 17th Ave, Aurora, CO 80045, USA; DAVID.ORMOND@CUANSCHUTZ.EDU; 4 Dell Medical School, Department of Oncology, The University of Texas Austin, 1701 Trinity Street, Austin, TX 78712, USA; gail.eckhardt@austin.utexas.edu

**Keywords:** triple-negative breast cancer, WEE1, 5-FU, DNA damage

## Abstract

Triple-negative breast cancer (TNBC) is an aggressive subtype defined by lack of hormone receptor expression and non-amplified HER2. Adavosertib (AZD1775) is a potent, small-molecule, ATP-competitive inhibitor of the Wee1 kinase that potentiates the activity of many DNA-damaging chemotherapeutics and is currently in clinical development for multiple indications. The purpose of this study was to investigate the combination of AZD1775 and capecitabine/5FU in preclinical TNBC models. TNBC cell lines were treated with AZD1775 and 5FU and cellular proliferation was assessed in real-time using IncuCyte^®^ Live Cell Analysis. Apoptosis was assessed via the Caspase-Glo 3/7 assay system. Western blotting was used to assess changes in expression of downstream effectors. TNBC patient-derived xenograft (PDX) models were treated with AZD1775, capecitabine, or the combination and assessed for tumor growth inhibition. From the initial PDX screen, two of the four TNBC PDX models demonstrated a better response in the combination treatment than either of the single agents. As confirmation, two PDX models were expanded for statistical comparison. Both PDX models demonstrated a significant growth inhibition in the combination versus either of the single agents. (TNBC012, *p* < 0.05 combo vs. adavosertib or capecitabine, TNBC013, *p* < 0.01 combo vs. adavosertib or capecitabine.) An enhanced anti-proliferative effect was observed in the adavosertib/5FU combination treatment as measured by live cell analysis. An increase in apoptosis was observed in two of the four cell lines in the combination when compared to single-agent treatment. Treatment with adavosertib as a single agent resulted in a decrease in p-CDC2 in a dose-dependent manner that was also observed in the combination treatment. An increase in γH2AX in two of the four cell lines tested was also observed. No significant changes were observed in Bcl-xL following treatment in any of the cell lines. The combination of adavosertib and capecitabine/5FU demonstrated enhanced combination effects both in vitro and in vivo in preclinical models of TNBC. These results support the clinical investigation of this combination in patients with TNBC, including those with brain metastasis given the CNS penetration of both agents.

## 1. Introduction

Triple-negative breast cancer (TNBC) is an aggressive breast cancer subtype defined by a lack of hormone receptor expression and non-amplified HER2 [1]. TNBC accounts for approximately 15% of breast cancer cases but is associated with an increased risk of cancer recurrence, brain metastasis, and death due to metastatic breast cancer [1]. Heterogeneity exists within TNBC. The luminal androgen receptor, mesenchymal, basal-like immunosuppressed and basal-like immune-activated subtypes have been described [2]. Mutations in p53 are common in TNBC, occurring in approximately 85% of tumors in the TCGA dataset [3]. While immunotherapy with the PD-L1-inhibitor atezolizumab prolongs progression-free survival in patients with PD-L1-positive TNBC when added to nab-paclitaxel, chemotherapy remains the standard treatment for metastatic disease and results in a median survival of 12–18 months [1]. There remains an unmet need for active targeted therapies in TNBC.

Adavosertib (AZD1775) is a potent, small-molecule, ATP-competitive inhibitor of the Wee1 and 2 kinases (*K*d 3.2 and 3.9 nM, respectively) [4] that potentiates the activity of many DNA-damaging chemotherapeutics and is currently in clinical development for multiple indications [5]. The Wee1 kinase is a key regulator of the G_2_ cell cycle checkpoint [6,7]. In response to DNA damage, Wee1 inhibits the activity of CDC2 through phosphorylation, resulting in cell cycle arrest to allow for DNA repair [6]. *TP53*-deficient cells with impaired G_1_ checkpoint function can be sensitized to DNA-damaging chemotherapeutics, with the addition of AZD1775 leading to abrogation of the G_2_ checkpoint [8]. AZD1775 can also potentiate the activity of DNA-damaging and antimetabolite chemotherapeutics in preclinical models without *TP53*-deficiency, possibly due to baseline replicative stress or compromised DNA repair proficiency [9,10]. AZD1775 enhances the cytotoxic effects of 5FU in *TP53*-deficient colon cancer cell lines and the 5FU prodrug capecitabine in xenograft models [5]. In these models, AZD1775 inhibited CDC2 Y15 phosphorylation and abrogated the G_2_ DNA damage checkpoint induced by 5FU, causing premature entry of mitosis. 

Capecitabine is an oral antimetabolic fluoropyrimidine deoxynucleoside carbamate drug that is converted to 5-fluorouracil in patients by thymidine phosphorylase, which then concentrates in the tumor tissue [11]. Capecitabine has been demonstrated to be effective in the adjuvant setting for patients with HER2-negative breast cancer who do not achieve a pathologic complete response following neoadjuvant chemotherapy and as a palliative therapy in metastatic TNBC [12,13,14,15]. Given the ability of AZD1775 to enhance the efficacy of DNA-damaging agents, 5-FU/capecitabine is a promising combination partner for AZD1775 in TNBC [5]. 

The purpose of this study was to evaluate rational combination partners for AZD1775 in preclinical models of TNBC using FDA-approved chemotherapies (5FU/capecitabine, paclitaxel, gemcitabine and doxorubicin) and other targeted agents available through the National Cancer Institute’s Cancer Therapy Evaluation Program (CTEP) (navitoclax, VX970, and romidepsin). Following the initial screen evaluating these combinations in TNBC PDX models, larger efficacy and mechanistic studies were performed for the combination of 5-FU/capecitabine and AZD1775.

## 2. Results

### 2.1. AZD1775 in Combination with Chemotherapy and Targeted Agents in TNBC PDX Models

**Figure 1:**
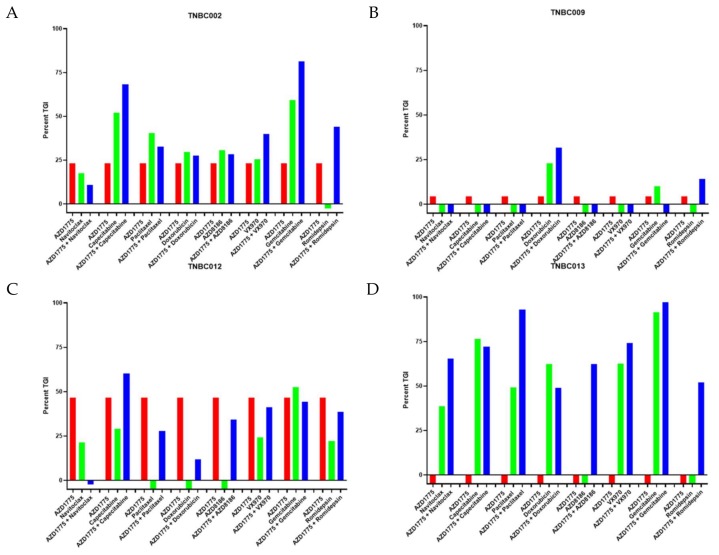
Effect of AZD1775 alone or in combination with chemotherapy or targeted agents in triple-negative breast cancer (TNBC) PDX models. Percent tumor growth inhibition (TGI) was calculated for each model (*n* = 3 animal/group). AZD1775, 50 mg/kg (PO, QD); paclitaxel, 15 mg/kg (IP, QW); capecitabine, 60 mg/kg (PO, QWx2); doxorubicin, 1.5 mg/kg (IP, QW); AZD8186, 25 mg/kg (IP, QD); navitoclax 100 mg/kg (PO QWx3); romidepsin 1.34 mg/kg (IP, QW); VX970, 40 mg/kg (IP, QW); gemcitabine 40 mg/kg (IP, QW). (**A**) TNBC002, (**B**) TNBC009, (**C**) TNBC012, and (**D**) TNBC013.

In an effort to minimize the number of animals required to evaluate multiple combinations with AZD1775, we performed an initial screen using four TNBC PDX models treated with AZD1775, capecitabine, paclitaxel, gemcitabine, doxorubicin, navitoclax (BCL-2 inhibitor), VX970 (ATR inhibitor), and romidepsin (HDAC inhibitor) or combinations of these agents with AZD1775. Each group consisted of three mice with two tumors each. As demonstrated in Figure 1, we observed an increased TGI with the combination of multiple agents with AZD1775 compared to any single agent alone. In particular, the combination of doxorubicin or paclitaxel and AZD1775 showed an enhanced combination effect in one PDX model each (TNBC009 and TNBC013, respectively). In addition, the combination of gemcitabine and AZD1775 was found to have a better combination effect in two out of the four models (TNBC0002 and TNBC013). However, due to this combination being actively researched in several clinical trials, we decided to not pursue this combination.

The combination of AZD1775 and capecitabine was identified for further evaluation based on an enhanced combination effect observed in two models (TNBC002, TNBC012) and the fact that both drugs cross the blood–brain barrier. Of note, in TNBC013, capecitabine as a single agent resulted in a very high TGI of approximately 75%, which may have limited the detection of a combination effect. 

### 2.2. AZD1775 in Combination with Capecitabine in PDX Models

**Figure 2:**
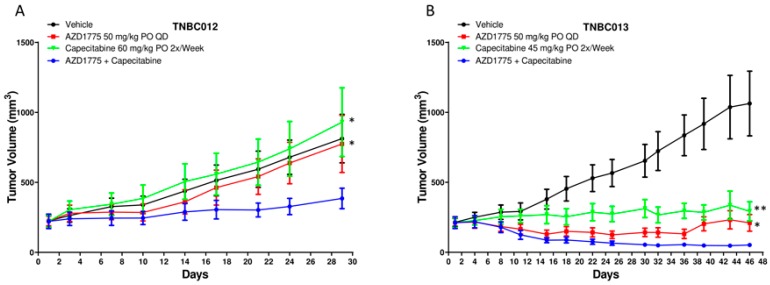
Effect of AZD1775 alone or in combination with capecitabine in TNBC PDX models (*n* = 10–12 animals/group). (**A**) TNBC012 and (**B**) TNBC013.

To confirm potentiation of the activity of AZD1775 with the addition of capecitabine in TNBC, we performed further in vivo studies using 2 TNBC PDX models (TNBC012 and TNBC013) with 5 animals (10 tumors) in each group. These models were selected for confirmation based on known p53 mutations and these tumors were isolated from brain metastasis in patients, which is relevant in TNBC given the high incidence of brain metastasis and CNS penetration of both agents. The dose of capecitabine was lowered in these experiments for TNBC013 based on the TGI observed with a higher dose in Figure 1. As depicted in Figure 2A,B, combination treatment resulted in a statistically significant tumor growth inhibition when compared with either single agent and tumor regression was observed in TNBC013 (Figure 2B).

### 2.3. Anti-Proliferative Effects of AZD1775 with 5FU in TNBC Cell Lines In Vitro

**Figure 3:**
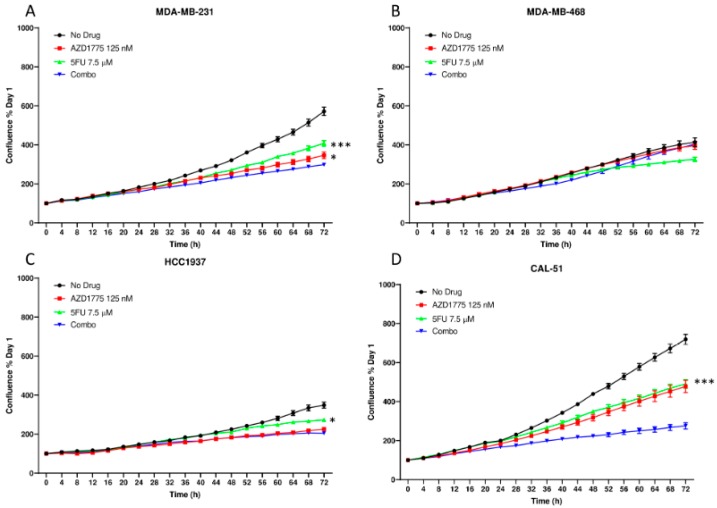
Anti-proliferative effects of AZD1775 and 5FU in TNBC cell lines. Cells were treated with AZD1775 and 5FU and proliferation was assessed for 72 h using the Incucyte Zoom and normalized to Day 1. (**A**) MDA-MB-231, (**B**) MDA-MB-468, (**C**) HCC1937, and (**D**) CAL-51. A t-test was performed to compare the combination treated to each of the single agent (* = *p* ≤ 0.05, *** = *p* ≤ 0.001).

**Figure 4:**
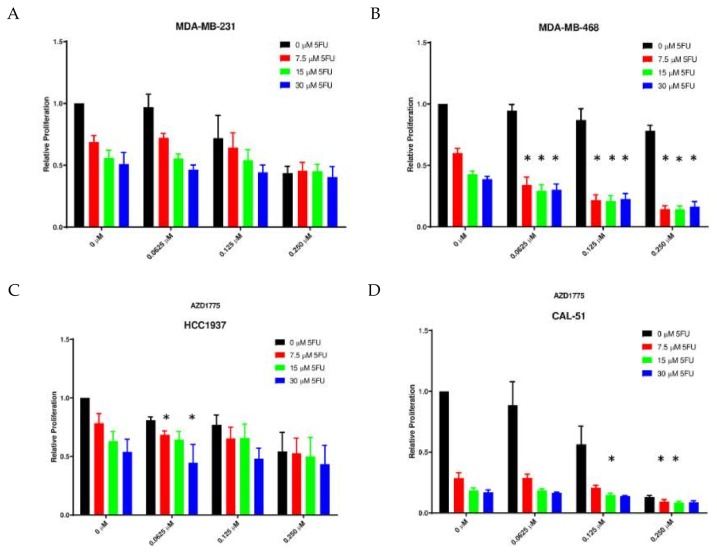
Cytotoxic effects of AZD1775 and 5FU in TNBC cell lines. Cells were treated with AZD1775 and 5FU and cytotoxic effects were assessed at 72 h using the sulforhodamine B (SRB) assay. Synergistic effects were analyzed using Calcusyn software. (**A**) MDA-MB-231, (**B**) MDA-MB-468, (**C**) HCC1937, and (**D**) CAL-51. (* = CI < 1).

Following confirmation of combination activity in vivo, we performed in vitro experiments to further characterize the anti-proliferative activity of the combination using live cell imaging and the sulforhodamine B (SRB) assay. We selected 5FU for use in vitro based on the required activation steps for the conversion of capecitabine to 5FU in vivo. We observed a statistically significant decrease in proliferation with the combination as compared to either AZD1775 and 5FU alone as assessed by live cell imaging in two of the four TNBC cell lines (MDA-MB-231 and CAL-51) (Figure 3A,D). In contrast, the HCC1937 only demonstrated an enhanced combination effect when compared to 5FU as a single agent and no combination effects were observed in the MDA-MB-468 TNBC cell line (Figure 3B,C). In the SRB assay, quantification of cellular proteins in cultured cells can be measured and synergistic responses can be calculated using the Chou and Talalay method. Using this assay, we observed synergistic combination effects in the MDA-MB-468, HCC1937, and CAL-51 cell lines at various concentrations or both drugs (* = CI < 1) (Figure 4).

### 2.4. Apoptotic Effects of AZD1775 with 5FU in TNBC Cell Lines

**Figure 5:**
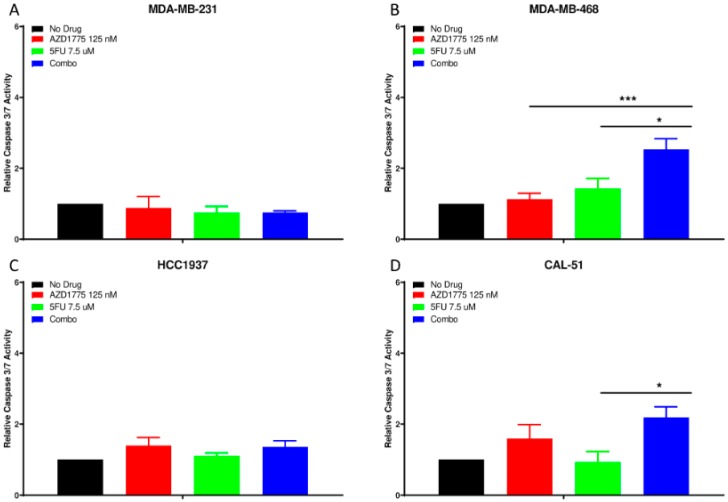
Apoptotic effects of AZD1775 and 5FU in TNBC cell lines. Cells were treated with AZD1775 and 5FU and caspase 3/7 activity was assessed at 24 h using Caspase Glo 3/7 assay. (**A**) MDA-MB-231, (**B**) MDA-MB-468, (**C**) HCC1937, and (**D**) CAL-51. A t-test was performed to compare the combination treated to each of the single agent (* = *p* ≤ 0.05; *** = *p* ≤ 0.001).

To determine the effect of AZD1775 and 5FU on apoptosis, the Caspase 3/7 assay was utilized. At 24 h, we observed no significant apoptosis in the MDA-MB-231 or HCC1937 cell lines with single agent or combination treatment (Figure 5A,C). However, there was a significant increase in apoptosis with the combination compared to both single agents in the MDA-MB-468 cell line (Figure 5B). In the CAL-51 TNBC cell line, apoptosis was only significantly higher in the combination compared to 5FU as a single agent (Figure 5D).

### 2.5. Cell Cycle Effects of AZD1775 with 5FU in TNBC Cell Lines

**Figure 6:**
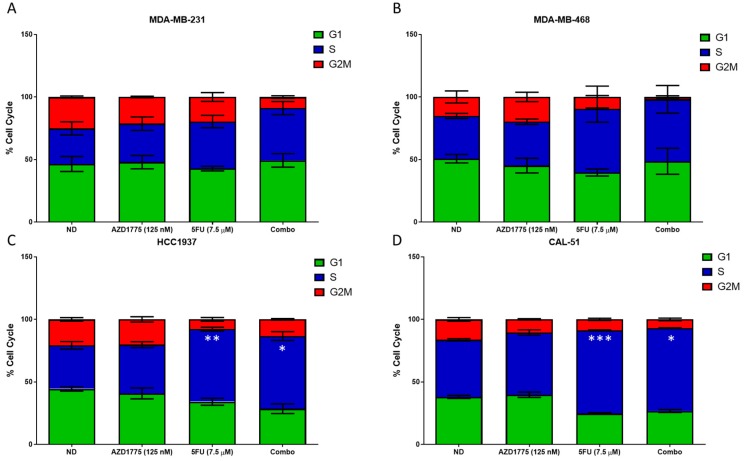
Cell cycle analysis of AZD1775 and 5FU in TNBC cell lines. Cells were treated with AZD1775 and 5FU and cell cycle arrest was assessed at 24 h using Krishans staining followed by flow cytometry. (**A**) MDA-MB-231, (**B**) MDA-MB-468, (**C**) HCC1937, and (**D**) CAL-51. A t-test was performed to compare the combination treated to each of the single agent (* = *p* ≤ 0.05, ** = *p* ≤ 0.01, **** = *p* ≤ 0.0001).

To determine the effects on the cell cycle, cells were exposed to AZD1775, 5FU or the combination for 24 h and analyzed by flow cytometry. As depicted in Figure 6A,B, there is no significant difference in cell cycle arrest in the MDA-MB-231 or MDA-MB-468 with any treatment. However, we did observe a slight decrease in G2/M in the combination compared to either single agent. In the HCC1937 cell line, there was a significant increase in S-phase arrest in 5FU treated single agent when compared to no drug (*p* < 0.01). Additionally, there was also a statistically significant increase in S-phase arrest in the combination, when compared to AZD1775 as a single agent (*p* < 0.05) (Figure 6C). Similarly, the CAL-51, demonstrated similar results as the HCC1937, with a statistically significant increase in S-phase arrest with 5FU as a single agent compared to no drug and the combination compared to AZD1775 as a single agent (*p* < 0.001 and *p* < 0.05, respectively) (Figure 6D).

### 2.6. Effects of AZD1775 with 5FU on Downstream Effectors

**Figure 7:**
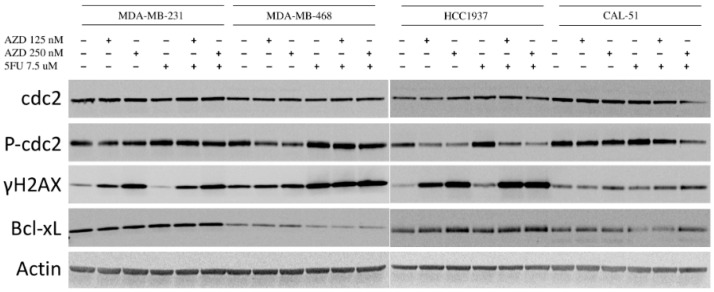
Effects of AZD1775 and 5FU on relevant downstream effectors of the cell cycle and DNA damage. Cells were treated with AZD1775 and 5FU for 48 h and total protein was extracted. Whole cell extracts were assessed for expression of CDC2. p-CDC2, γH2AX, and Bcl-xL by immunoblotting.

Immunoblotting was performed to elucidate the mechanism of the combination effects of AZD1775 with 5FU in the four TNBC cell lines. Following 48 h exposure to the drugs, two of the four cell lines (MDA-MB-468 and HCC1937) demonstrated a decrease in p-CDC2 in a dose-dependent manner with AZD1775 as a single agent, and the decrease was only maintained in the combinations for the HCC1937 cell line. An increase in in γ-H2AX was observed in the AZD1775 single agent at both dose levels in the MDA-MB-231 and the HCC1937 and was enhanced in the 125 nM combo for the HCC1937. In the MDA-MB-48 and CAL51, no increase in γ-H2AX was observed in either AZD1775 as a single agent. However, there was an enhanced increase in the 250 nM combination. A decrease in Bcl-xL was observed with the 5FU and combination treatment in the MDA-MB-468 and CAL-51 cell lines, indicating an increase in apoptosis (Figure 7 and Supplementary Data).

## 3. Discussion

Approximately 15% of breast cancers in the United States are classified as TNBC and, despite recent advances in local and systemic therapies, patients with TNBC continue to be at increased risk of metastatic recurrence and have inferior outcomes compared to other breast cancer subtypes [16]. Therefore, many new and novel targeted therapies are being explored for the treatment of TNBC. AZD1775 is an inhibitor of WEE1 kinase, which is an inhibitory regulator of the G2/M checkpoint by phosphorylating and inactivating CDC2. The G2/M arrest allows tumor cells time to repair any damage. By inhibiting WEE1, cells progress through the G2/M checkpoint and die via mitotic catastrophe. Since tumors with defective p53 rely on the G2/M checkpoint, it is thought that by abrogating this checkpoint with a WEE1 inhibitor, it may preferentially sensitize the p53-mutated TNBC to DNA-damaging agents [17]. In the current study, we performed an in vivo screen of chemotherapies and targeted agents from the CTEP portfolio available for investigator-initiated preclinical and clinical trials as single agents and in combination with the WEE1 inhibitor AZD1775. In this screen, we discovered that AZD1775 in combination with capecitabine demonstrated enhanced anti-tumor effects compared to either single agent alone. We then further validated this combination in additional TNBC PDX models and TNBC cell lines to characterize the mechanism of the synergy. 

Our study demonstrates that the addition of AZD1775 to capecitabine enhanced the anti-tumor effects in additional TNBC PDX models and TNBC cell lines. We observed an increase in apoptosis in several cell lines with the addition of AZD1775 to either agent. AZD1775 treatment led to a decrease in p-CDC2 and an increase γ-H2AX that was either maintained or enhanced in the combination with capecitabine demonstrating a DNA damage response. This is similar to what was observed in pancreatic cancer cell lines where gemcitabine, when combined with AZD1775 increased the amount of γ-H2AX staining by flow cytometry [18]. In fact, the authors went further and determined that γ-H2AX staining pattern may be a marker of sensitization. 

It is thought that the addition of AZD1775 to DNA-damaging agents like chemotherapies may be a viable treatment strategy for various tumor types. Hirai et al. demonstrated that combining AZD1775 with gemcitabine, 5FU, capecitabine, and irinotecan enhanced the effects of each of these chemotherapies in colon and breast cancer models [5]. The authors tested the combination of 5FU and AZD1775 in p53 WT colon cancer models and did not see any enhancement in cell viability. This is in contrast to what was observed in our work, in that enhanced anti-proliferative effects were observed in the p53 WT, Cal-51 cell line, as well p53 MT TNBC cell lines. In the current study, not all cell lines responded similarly in all assays perhaps due to fundamental differences in the endpoints of the various assays or inherent genetic differences other than p53. These differences in response could also explain varying responses in the genetic makeup of other tumor types. AZD1775 has also been shown to enhance the effect of cisplatin in gastric cancer models. When gastric cancer cell lines were exposed to AZD1775 and cisplatin, an enhanced anti-proliferative effect was observed and an increase in apoptosis [19]. Similar to what we observed, the authors demonstrated that the combination worked in both p53 WT and p53 MT gastric cancer cell lines. However, the combination effect was better in the p53 MT. In addition to chemotherapy combinations, AZD1775 has shown efficacy when combined with other small molecules. Olaparib, in combination with AZD1775, demonstrated enhanced anti-tumor effects in both small cell lung and ovarian cancers [20,21]. These studies demonstrated a synergistic anti-proliferative effect, and an enhancement in apoptosis and decreases in p-CDC2 in AZD1775-treated ovarian cancer cells that was maintained in the combination. Additionally, AZD1775 in combination with the Aurora Kinase A inhibitor, alisertib, also demonstrated an enhanced anti-tumor effect with increases in apoptosis and γ-H2AX in head and neck cancers [22]. These data suggest that combination therapies with AZD1775 and chemotherapy or other targeted agents are viable options for treatment for various tumor types.

Several clinical trials have been conducted or are currently ongoing evaluating AZD1775 as a single agent and in combination with other cancer therapies. In a phase I trial of AZD1775 in patients with refractory solid tumors, the compound was tolerable with myelosuppression as the main dose-limiting toxicity. Partial responses were observed in two patients with *BRCA1* mutations and no difference in response between patients with p53 mutations and wild-type p53 was observed. In this trial, post treatment tumor biopsies demonstrated a decrease in p-CDC2 and an increase in γ-H2AX which is consistent with our observations preclinically [23]. In a phase I clinical trial evaluating AZD1775 in combination with gemcitabine, cisplatin, or carboplatin in patients with advanced solid tumors, the most common adverse events included nausea, vomiting, diarrhea, and hematologic toxicity [24]. Stable disease was observed in 53% of patients and 10% achieved partial response. There was a trend towards an improved objective response rate in patients with p53 mutations compared to wild-type p53 in this trial (21% vs. 12%). 

Further adding to the promise of AZD1775 as a cancer therapeutic is documented CNS penetration, which is particularly interesting in TNBC with its high incidence of *TP53* mutations and brain metastasis [25,26]. CNS penetration by AZD1775 was confirmed by Li et al. using plasma and brain tumor samples from patients with glioblastoma for pharmacokinetic analysis [25]. Penetration of the human blood–brain barrier by AZD1775 is facilitated by uptake into the CNS through the OATP1A2 transporter and limited transporter-mediated efflux by ABCB1/ABCG2 in the relatively acidic tumor microenvironment. The result is favorable CNS penetration into brain tumors [25]. Capecitabine is a promising combination partner for AZD1775 due to non-overlapping dose-limiting toxicities (DLTs), CNS penetration and activity in TNBC in the adjuvant and metastatic setting [27,28,29].

## 4. Materials and Methods

### 4.1. TNBC Patient-Derived Xenografts

Patient-derived tumors were acquired from the University of Colorado Hospital (Aurora, CO, USA) following consent and in accordance with protocols approved by the Colorado Multiple Institutional Review Board. Female athymic nude mice (4–8 weeks of age) were purchased from Envigo (Indianapolis, IN, USA). All animal studies were approved by the Institutional Animal Care and Use Committee (00021). Tumor specimens were implanted into the hind flanks of mice and maintained for several generations as described previously [30]. For treatment studies, tumors were expanded into the hind flanks of mice when the tumors reached 150–300 mm^3^, and were randomized into treatment groups as previously described [31,32,33]. For the initial screen, mice (*n* = 3/group) were treated with AZD1775 alone or in combination with capecitabine, paclitaxel, gemcitabine, doxorubicin, navitoclax, VX970, and romidepsin. In the expanded cohorts, mice (*n* = 10/group) were treated with AZD1775 (50 mg/kg, PO, QD, AstraZeneca, Cambridge, MA, USA), or capecitabine (60 mg/kg, PO, twice weekly), as single agents or in combination as described previously [34,35]. Mice were monitored daily for signs of toxicity and tumor size was evaluated using digital calipers twice weekly using the following formula: tumor volume = (length × width^2^) × 0.52. Percent tumor growth inhibition (TGI) values were calculated using the following formula: ((MTV_vehicle_ − MTV_treated_)/MTV_vehicle_) × 100.

### 4.2. Drugs

AZD1775 was provided by AstraZeneca or purchased from MolPool (Kowloon, Hong Kong). Capecitabine was purchased from Active Biochem (Kowloon, Hong Kong). The 5-fluorouracil was purchased from the University of Colorado Hospital Pharmacy (Aurora, CO, USA). 

### 4.3. Cell Lines and Reagents

HCC1937, CAL-51, MDA-MB-231, and MDA-MB-468 were purchased from ATCC and maintained in DMEM (Corning) containing 10% FBS (Atlas Biologicals, Ft. Collins, CO, USA) with 1% Pen/Strep and 1% non-essential amino acids (Corning, Tewksbury, MA, USA). Cells were routinely tested for mycoplasma and were authenticated at the Barbara Davis Center for Diabetes Molecular Biology Service Center (Aurora, CO, USA). 

### 4.4. Proliferation and Apoptosis

The anti-proliferative effects of AZD1775 in combination with 5FU were evaluated on HCC1937, CAL-51, MDA-MB-231, and MDA-MB-468 using the IncuCyte Zoom live cell imager (Essen Bioscience, Ann Arbor, MI, USA). All cell lines were plated in 96-well plates at optimal density and allowed to adhere for 24 h. After 24 h, the cells were treated with AZD1775 (125 or 250 nM) and 5-fluorouracil (5FU) (7.5 µM), as single agents or in combination. Doses of each compound were based on previously published data [36,37]. Plates were then placed in the IncuCyte Zoom and allowed to incubate for 72 h while taking images every 2–4 h. Following 72 h, the plates were removed and discarded. Percent confluence was analyzed over time using the IncuCyte Zoom 2018A software (Essen Bioscience, Ann Arbor, MI, USA) and graphed as a measure of percent confluence over time with GraphPad Prism 8.1 (San Diego, CA, USA). To assess cell cytotoxicity, the sulforhodamine B (SRB) assay was performed and synergy was calculated using the Calcysyn software (Biosoft, v2, company, Campridge, UK) as described previously [33,38]. A CI value of <1 was considered synergistic. For analysis of apoptosis, all cell lines were plated in white-walled 96-well plates and allowed to adhere for 24 h. After 24 h, the cells were treated as described above for 24 h. Apoptosis was assessed using the Caspase Glo 3/7 assay (Promega,Madison, WI, USA) per manufacturer instructions and luminescence was measured using the Synergy H1 plate reader (BioTek, Winooski, VT, USA). Relative caspase activity was calculated and graphed using GraphPad Prism 8.1. 

### 4.5. Cell Cycle Analysis

The effect of AZD1775 in combination with 5FU on cell cycle was evaluated in HCC1937, CAL-51, MDA-MB-231, and MDA-MB-468 cell lines. All cell lines were plated in 6-well plates at optimal density and allowed to adhere for 24 h. After 24 h, the cells were treated with AZD1775 (125 or 250 nM) and 5FU (7.5 µM), as single agents or in combination for an additional 24 h. Cells were then trypsinized, washed with PBS and resuspended in Krishan’s stain and stored at 4 °C overnight. Cell cycle distribution was analyzed by flow cytometry in the University of Colorado Cancer Center Flow Cytometry Core (Aurora, CO, USA). 

### 4.6. Immunoblotting

The effects of drug treatments on downstream effectors were assessed by immunoblotting. Cells were plated in a 6-well plate and allowed to adhere for 24 h. After 24 h, the cells were treated with AZD1775 (125 or 250 nM) or 5-fluorouracil (5FU) (7.5µM), as single agents or in combination for 24 h. Following treatment, lysis buffer containing protease and phosphatase inhibitors were added to the wells and the cells were scraped on ice. The lysed proteins were added to a microcentrifuge tube and sonicated for 30 s. Following sonication, the lysates were centrifuged for 10 minutes at 16,000× *g* at 4 °C. The supernatant was removed and transferred to a new tube and protein concentration was determined using a Pierce BCA Protein Assay Kit (Thermo Fisher, Waltham, MA, USA). A total of 40 µg of protein was electrophoresed on a precast 4–12% BisTris gel (Life Technologies, Carlsbad, CA, USA). Proteins were then transferred onto a nitrocellulose membrane using Invitrogen Power Blotter (Thermo Fisher). Membranes were blocked for 1 h at room temperature using 3% casein. After blocking, the membranes were probed with the following primary antibodies (1:1000) overnight at 4 °C with rocking: CDC2, p-CDC2, H2AX, Bcl-xL, and Actin. Following washing three times with TBST, the membranes were incubated for 1 h at room temperature with anti-rabbit or -mouse IgG (H+L; DyLight 800 or 600) conjugated sary antibody at a dilution of 1:15,000. Images were captured using the Odyssey Infrared Imaging System (Licor, Lincoln, New, USA). (All original Western blot figures can be found in the Supplementary Date.)

### 4.7. Statistical Analysis

We compared combination treatment groups to each of the single agents and performed an unpaired *t*-test with Welch’s correction GraphPad Prism 8.0, (San Diego, CA, USA).

## 5. Conclusions

The combination of AZD1775 with capecitabine leads to enhanced anti-proliferative effects in vitro and in vivo. Enhanced apoptosis and cell cycle arrest was also observed. Additionally, an increase in the DNA damage response was observed by immunoblotting in the AZD1775 single agent and this was maintained in the combination. This work supports the future investigation of the combination of AZD1775 and capecitabine in patients with metastatic TNBC.

## Supplementary Materials

The following are available online at https://www.mdpi.com/2072-6694/12/3/719/s1, Supplementary Date: All original Western blot figures.

## References

[1] Dent R., Trudeau M., Pritchard K.I., Hanna W.M., Kahn H.K., Sawka C.A., Lickley L.A., Rawlinson E., Sun P., Narod S.A. (2007). Triple-negative breast cancer: Clinical features and patterns of recurrence. Clin. Cancer Res..

[2] Burstein M.D., Tsimelzon A., Poage G.M., Covington K.R., Contreras A., Fuqua S.A., Savage M.I., Osborne C.K., Hilsenbeck S.G., Chang J.C. (2015). Comprehensive genomic analysis identifies novel subtypes and targets of triple-negative breast cancer. Clin. Cancer Res..

[3] Kandoth C., McLellan M.D., Vandin F., Ye K., Niu B., Lu C., Xie M., Zhang Q., McMichael J.F., Wyczalkowski M.A. (2013). Mutational landscape and significance across 12 major cancer types. Nature.

[4] Wright G., Golubeva V., Remsing Rix L.L., Berndt N., Luo Y., Ward G.A., Gray J.E., Schonbrunn E., Lawrence H.R., Monteiro A.N.A. (2017). Dual Targeting of WEE1 and PLK1 by AZD1775 Elicits Single Agent Cellular Anticancer Activity. ACS Chem. Biol..

[5] Hirai H., Arai T., Okada M., Nishibata T., Kobayashi M., Sakai N., Imagaki K., Ohtani J., Sakai T., Yoshizumi T. (2010). MK-1775, a small molecule Wee1 inhibitor, enhances anti-tumor efficacy of various DNA-damaging agents, including 5-fluorouracil. Cancer Biol. Ther..

[6] Russell P., Nurse P. (1987). Negative regulation of mitosis by wee1+, a gene encoding a protein kinase homolog. Cell.

[7] Aarts M., Linardopoulos S., Turner N.C. (2013). Tumour selective targeting of cell cycle kinases for cancer treatment. Curr. Opin. Pharmacol..

[8] Kogiso T., Nagahara H., Hashimoto E., Ariizumi S., Yamamoto M., Shiratori K. (2014). Efficient induction of apoptosis by wee1 kinase inhibition in hepatocellular carcinoma cells. PLoS ONE.

[9] Aarts M., Bajrami I., Herrera-Abreu M.T., Elliott R., Brough R., Ashworth A., Lord C.J., Turner N.C. (2015). Functional Genetic Screen Identifies Increased Sensitivity to WEE1 Inhibition in Cells with Defects in Fanconi Anemia and HR Pathways. Mol. Cancer Ther..

[10] Pfister S.X., Markkanen E., Jiang Y., Sarkar S., Woodcock M., Orlando G., Mavrommati I., Pai C.C., Zalmas L.P., Drobnitzky N. (2015). Inhibiting WEE1 Selectively Kills Histone H3K36me3-Deficient Cancers by dNTP Starvation. Cancer Cell.

[11] Miwa M., Ura M., Nishida M., Sawada N., Ishikawa T., Mori K., Shimma N., Umeda I., Ishitsuka H. (1998). Design of a novel oral fluoropyrimidine carbamate, capecitabine, which generates 5-fluorouracil selectively in tumours by enzymes concentrated in human liver and cancer tissue. Eur. J. Cancer.

[12] Blum J.L., Dieras V., Lo Russo P.M., Horton J., Rutman O., Buzdar A., Osterwalder B. (2001). Multicenter, Phase II study of capecitabine in taxane-pretreated metastatic breast carcinoma patients. Cancer.

[13] Blum J.L., Jones S.E., Buzdar A.U., LoRusso P.M., Kuter I., Vogel C., Osterwalder B., Burger H.U., Brown C.S., Griffin T. (1999). Multicenter phase II study of capecitabine in paclitaxel-refractory metastatic breast cancer. J. Clin. Oncol..

[14] Miles D., Vukelja S., Moiseyenko V., Cervantes G., Mauriac L., Van Hazel G., Liu W.Y., Ayoub J.P., O’Shaughnessy J.A. (2004). Survival benefit with capecitabine/docetaxel versus docetaxel alone: Analysis of therapy in a randomized phase III trial. Clin. Breast Cancer.

[15] O’Shaughnessy J., Miles D., Vukelja S., Moiseyenko V., Ayoub J.P., Cervantes G., Fumoleau P., Jones S., Lui W.Y., Mauriac L. (2002). Superior survival with capecitabine plus docetaxel combination therapy in anthracycline-pretreated patients with advanced breast cancer: Phase III trial results. J. Clin. Oncol..

[16] Perou C.M., Sorlie T., Eisen M.B., van de Rijn M., Jeffrey S.S., Rees C.A., Pollack J.R., Ross D.T., Johnsen H., Akslen L.A. (2000). Molecular portraits of human breast tumours. Nature.

[17] Leijen S., Beijnen J.H., Schellens J.H. (2010). Abrogation of the G2 checkpoint by inhibition of Wee-1 kinase results in sensitization of p53-deficient tumor cells to DNA-damaging agents. Curr. Clin. Pharmacol..

[18] Parsels L.A., Parsels J.D., Tanska D.M., Maybaum J., Lawrence T.S., Morgan M.A. (2018). The contribution of DNA replication stress marked by high-intensity, pan-nuclear gammaH2AX staining to chemosensitization by CHK1 and WEE1 inhibitors. Cell Cycle.

[19] Chen D., Lin X., Gao J., Shen L., Li Z., Dong B., Zhang C., Zhang X. (2018). Wee1 Inhibitor AZD1775 Combined with Cisplatin Potentiates Anticancer Activity against Gastric Cancer by Increasing DNA Damage and Cell Apoptosis. Biomed. Res. Int..

[20] Lallo A., Frese K.K., Morrow C.J., Sloane R., Gulati S., Schenk M.W., Trapani F., Simms N., Galvin M., Brown S. (2018). The Combination of the PARP Inhibitor Olaparib and the WEE1 Inhibitor AZD1775 as a New Therapeutic Option for Small Cell Lung Cancer. Clin. Cancer Res..

[21] Meng X., Bi J., Li Y., Yang S., Zhang Y., Li M., Liu H., Li Y., McDonald M.E., Thiel K.W. (2018). AZD1775 Increases Sensitivity to Olaparib and Gemcitabine in Cancer Cells with p53 Mutations. Cancers.

[22] Lee J.W., Parameswaran J., Sandoval-Schaefer T., Eoh K.J., Yang D.H., Zhu F., Mehra R., Sharma R., Gaffney S.G., Perry E.B. (2019). Combined Aurora Kinase A (AURKA) and WEE1 Inhibition Demonstrates Synergistic Antitumor Effect in Squamous Cell Carcinoma of the Head and Neck. Clin. Cancer Res..

[23] Do K., Wilsker D., Ji J., Zlott J., Freshwater T., Kinders R.J., Collins J., Chen A.P., Doroshow J.H., Kummar S. (2015). Phase I Study of Single-Agent AZD1775 (MK-1775), a Wee1 Kinase Inhibitor, in Patients with Refractory Solid Tumors. J. Clin. Oncol..

[24] Leijen S., van Geel R.M., Pavlick A.C., Tibes R., Rosen L., Razak A.R., Lam R., Demuth T., Rose S., Lee M.A. (2016). Phase I Study Evaluating WEE1 Inhibitor AZD1775 As Monotherapy and in Combination with Gemcitabine, Cisplatin, or Carboplatin in Patients with Advanced Solid Tumors. J. Clin. Oncol..

[25] Li J., Wu J., Bao X., Honea N., Xie Y., Kim S., Sparreboom A., Sanai N. (2017). Quantitative and Mechanistic Understanding of AZD1775 Penetration across Human Blood-Brain Barrier in Glioblastoma Patients Using an IVIVE-PBPK Modeling Approach. Clin. Cancer Res..

[26] Anders C.K., Carey L.A. (2009). Biology, metastatic patterns, and treatment of patients with triple-negative breast cancer. Clin. Breast Cancer.

[27] Morikawa A., Peereboom D.M., Thorsheim H.R., Samala R., Balyan R., Murphy C.G., Lockman P.R., Simmons A., Weil R.J., Tabar V. (2015). Capecitabine and lapatinib uptake in surgically resected brain metastases from metastatic breast cancer patients: A prospective study. Neuro-Oncology.

[28] Kaufman P.A., Awada A., Twelves C., Yelle L., Perez E.A., Velikova G., Olivo M.S., He Y., Dutcus C.E., Cortes J. (2015). Phase III open-label randomized study of eribulin mesylate versus capecitabine in patients with locally advanced or metastatic breast cancer previously treated with an anthracycline and a taxane. J. Clin. Oncol..

[29] Dranitsaris G., Gluck S., Faria C., Cox D., Rugo H. (2015). Comparative effectiveness analysis of monotherapy with cytotoxic agents in triple-negative metastatic breast cancer in a community setting. Clin. Ther..

[30] Bagby S., Messersmith W.A., Pitts T.M., Capasso A., Varella-Garcia M., Klauck P.J., Kim J., Tan A.C., Eckhardt S.G., Tentler J.J. (2016). Development and Maintenance of a Preclinical Patient Derived Tumor Xenograft Model for the Investigation of Novel Anti-Cancer Therapies. J. Vis. Exp..

[31] Capasso A., Pitts T.M., Klauck P.J., Bagby S.M., Westbrook L., Kaplan J., Soleimani M., Spreafico A., Tentler J.J., Diamond J.R. (2018). Dual compartmental targeting of cell cycle and angiogenic kinases in colorectal cancer models. Anticancer Drugs.

[32] Ionkina A.A., Tentler J.J., Kim J., Capasso A., Pitts T.M., Ryall K.A., Howison R.R., Kabos P., Sartorius C.A., Tan A.C. (2017). Efficacy and Molecular Mechanisms of Differentiated Response to the Aurora and Angiogenic Kinase Inhibitor ENMD-2076 in Preclinical Models of p53-Mutated Triple-Negative Breast Cancer. Front. Oncol..

[33] Schreiber A.R., Nguyen A., Bagby S.M., Arcaroli J.J., Yacob B.W., Quackenbush K., Guy J.L., Crowell T., Stringer B., Danaee H. (2018). Evaluation of TAK-264, an Antibody-Drug Conjugate in Pancreatic Cancer Cell Lines and Patient-Derived Xenograft Models. Clin. Cancer Drugs.

[34] Naughton M. (2010). Evolution of capecitabine dosing in breast cancer. Clin. Breast Cancer.

[35] Pokorny J.L., Calligaris D., Gupta S.K., Iyekegbe D.O., Mueller D., Bakken K.K., Carlson B.L., Schroeder M.A., Evans D.L., Lou Z. (2015). The Efficacy of the Wee1 Inhibitor MK-1775 Combined with Temozolomide Is Limited by Heterogeneous Distribution across the Blood-Brain Barrier in Glioblastoma. Clin. Cancer Res..

[36] Jin J., Fang H., Yang F., Ji W., Guan N., Sun Z., Shi Y., Zhou G., Guan X. (2018). Combined Inhibition of ATR and WEE1 as a Novel Therapeutic Strategy in Triple-Negative Breast Cancer. Neoplasia.

[37] Takahashi K., Tanaka M., Inagaki A., Wanibuchi H., Izumi Y., Miura K., Nagayama K., Shiota M., Iwao H. (2013). Establishment of a 5-fluorouracil-resistant triple-negative breast cancer cell line. Int. J. Oncol..

[38] Tentler J.J., Ionkina A.A., Tan A.C., Newton T.P., Pitts T.M., Glogowska M.J., Kabos P., Sartorius C.A., Sullivan K.D., Espinosa J.M. (2015). p53 Family Members Regulate Phenotypic Response to Aurora Kinase A Inhibition in Triple-Negative Breast Cancer. Mol. Cancer Ther..

