# Supplementary material for: Wee1 Inhibition Enhances the Anti-Tumor Effects of Capecitabine in Preclinical Models of Triple-Negative Breast Cancer
Source: Cancers (Basel). 2020 Mar 19;12(3):719. doi: 10.3390/cancers12030719 (PMC7140086; doi:10.3390/cancers12030719)
Supplement: Supplementary file 1 [file cancers-12-00719-s001.pdf]

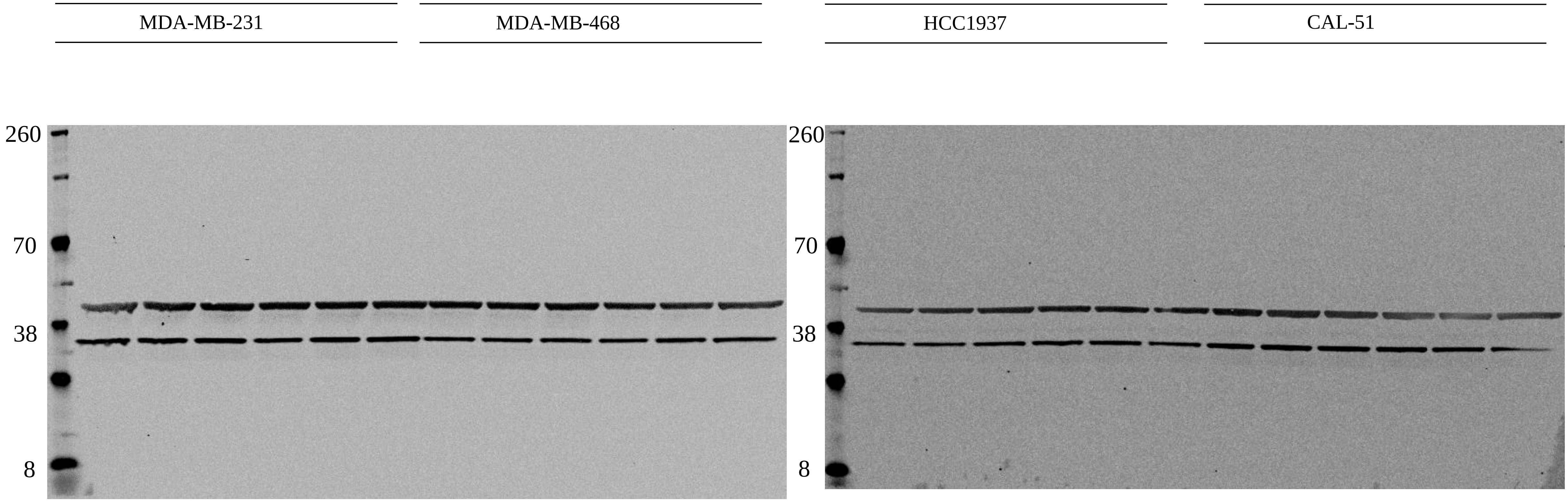

actin	33667:41244:45648:41305:41212:42261:40484:40405:39891:33274:30364:29251	32880:37259:40890:42069:42503:38794:44615:46706:44462:38092:32168:37805
cdc2	59657:73705:91660:61045:91169:88277:49793:45410:46723:38678:48457:45831	17102:17358:26799:27925:29496:20333:49783:56309:48389:48344:33368:13870

MDA-MB-231 | MDA-MB-468 | HCC1937 | CAL-51

260
70
38
8

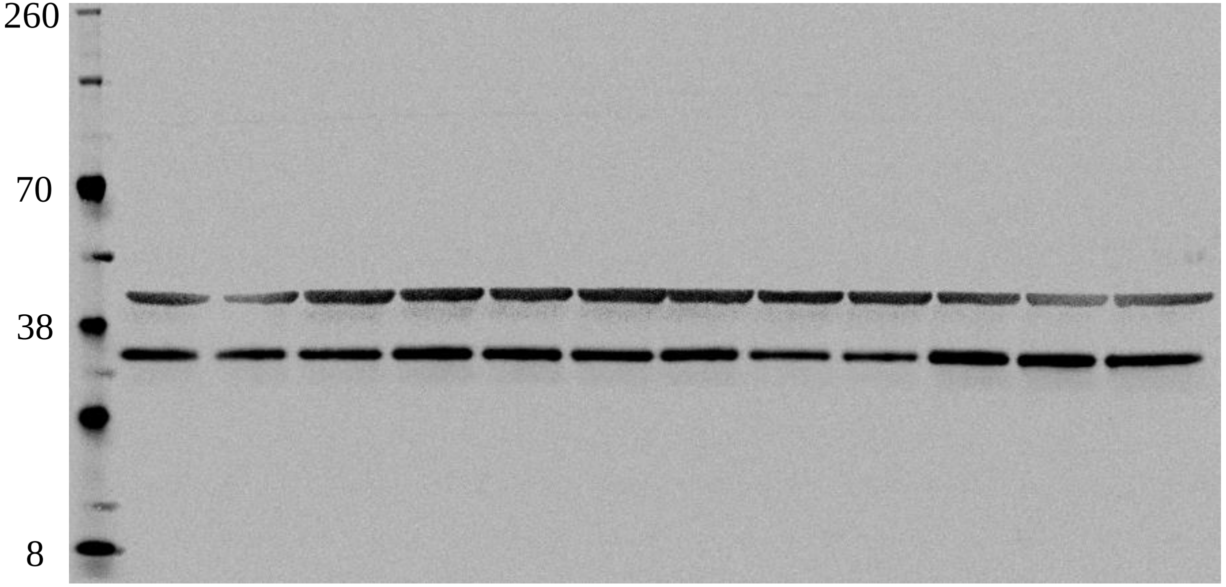

260
70
38
8

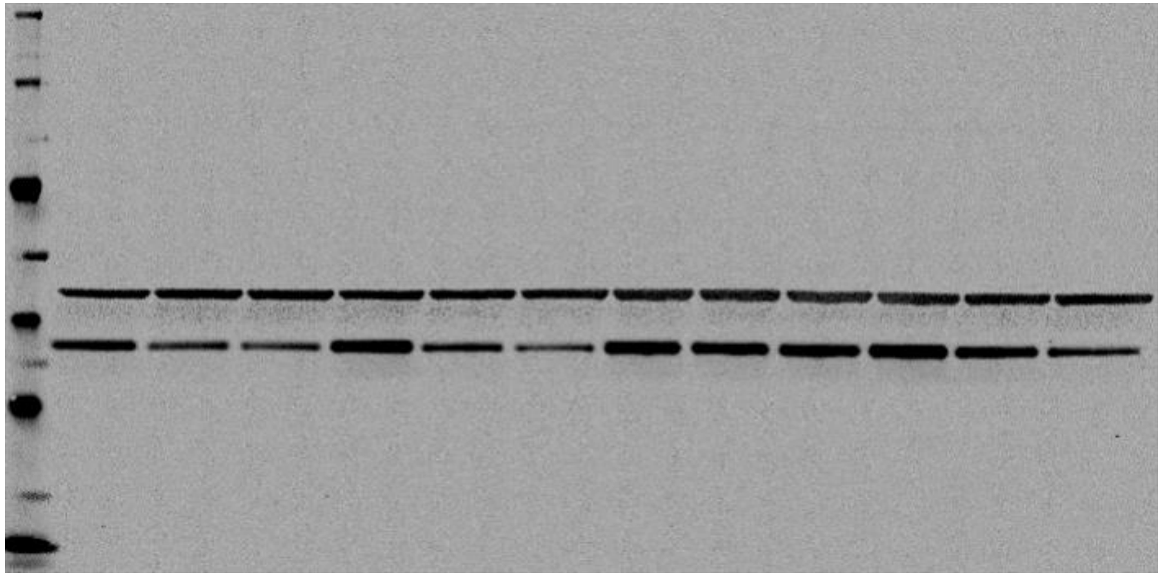

actin 23802:14896:30692:31724:28719:32009:29800:30789:28063:24096:16729:20904 20067:23416:23734:20672:20962:19273:18447:18824:17202:18628:20465:20725

P-cdc2 67413:50928:73119:100945:95664:84825:86917:51600:45333:109972:106713:93783 55694:30034:26989:102404:39533:22915:91601:79167:83784:117043:82241:38968

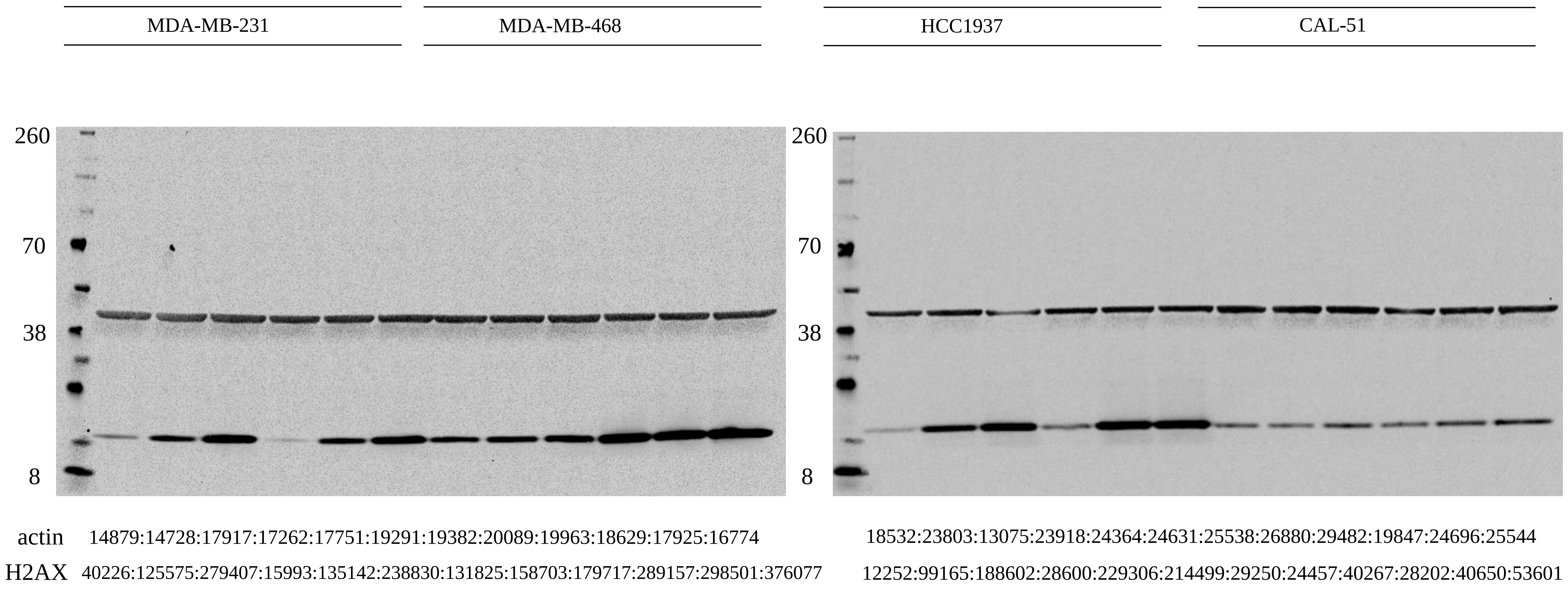

MDA-MB-231
MDA-MB-468
HCC1937
CAL-51
260
70
38
8
260
70
38
8
actin
14879:14728:17917:17262:17751:19291:19382:20089:19963:18629:17925:16774
18532:23803:13075:23918:24364:24631:25538:26880:29482:19847:24696:25544
H2AX
40226:125575:279407:15993:135142:238830:131825:158703:179717:289157:298501:376077
12252:99165:188602:28600:229306:214499:29250:24457:40267:28202:40650:53601

MDA-MB-231	MDA-MB-468	HCC1937	CAL-51

260
70
38
8

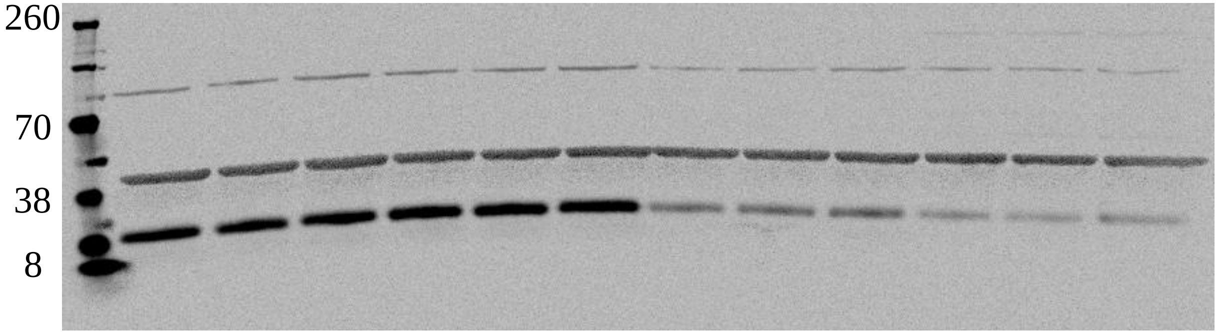

260
70
38
8

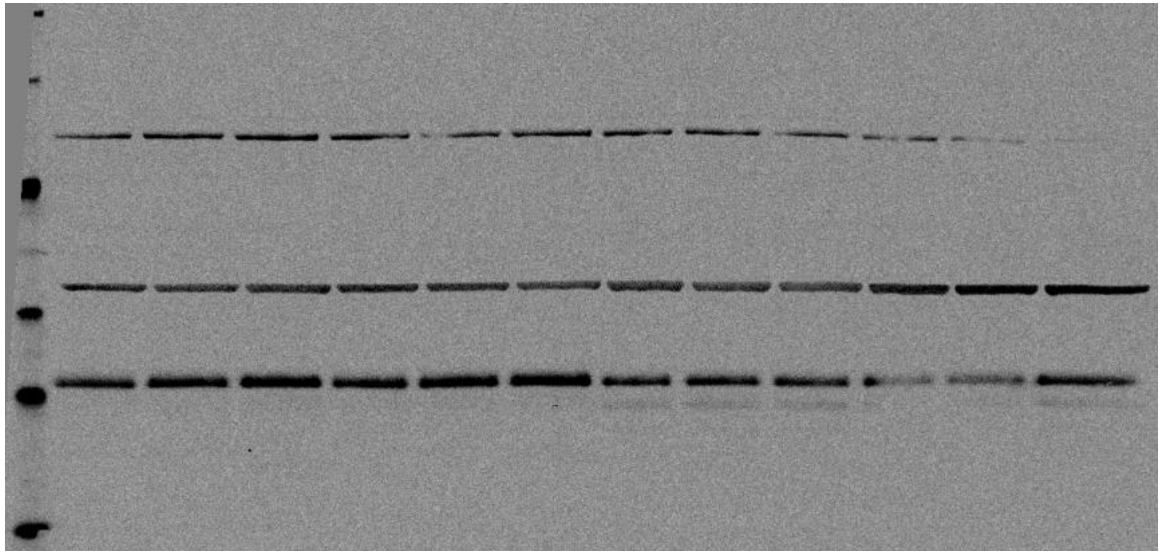

actin 14504:17192:14605:14543:13525:15068:12924:17448:13495:18357:17214:15363 15016:15603:18703:18162:16981:15708:17546:16490:17141:22031:25800:25611

Bcl-XL 73153:63027:72540:83711:82015:86053:21536:24087:26001:11707:9479:12561 19239:23876:32046:23777:28350:33143:16579:19140:16128:7777:9886:20001
